# Physical, Emotional, Medical, and Socioeconomic Status of Patients With NMOSD: A Cross-Sectional Survey of 123 Cases From a Single Center in North China

**DOI:** 10.3389/fneur.2021.737564

**Published:** 2021-09-08

**Authors:** Zhen Jia, XiuYu Dong, Shuang Song, Ruoyi Guo, Lu Zhang, Jia Liu, Bin Li

**Affiliations:** ^1^Department of Neurology, The Second Hospital of Hebei Medical University, Shijiazhuang, China; ^2^Neurological Laboratory of Hebei, Shijiazhuang, China; ^3^Department of Neurology, Dongzhimen Hospital, Beijing University of Chinese Medicine, Beijing, China; ^4^Institute for Brain Disorders, Beijing University of Chinese Medicine, Beijing, China

**Keywords:** neuromyelitis optica spectrum disorder, survival status, negative emotions, questionnaire survey, qualitative research

## Abstract

**Objective:** This study aimed to assess the physical, emotional, medical, and socioeconomic conditions of patients with neuromyelitis optica spectrum disorder (NMOSD) in North China.

**Methods:** A cross-sectional survey of patients with NMOSD was performed, based on an established questionnaire from the Multiple Sclerosis Patient Survival Report 2018. Logistic regression analysis was conducted to define the significant determinants of certain physical or emotional characteristics of patients. A total of 123 patients were included.

**Results:** A total of 63.4% of participants were initially diagnosed with conditions other than NMOSD, with a median delay of 6 months for accurate diagnosis. An aggregate of 72.2% of patients had one or more relapses, corresponding to an annual relapse rate of 0.8. Paresthesia was the most frequent physical symptom among patients both at disease onset (53.7%) and throughout the duration of the disease (86.2%). Onset in elderly (>50 years) patients was associated with an annual Expanded Disability Status Scale increase ≥1, compared with onset in younger (<30 years) patients (*P* = 0.001, OR = 7.83). A total of 76.4% of patients had received attack-prevention treatments in the remission phase, and 31.7 and 10.6% of patients had ever been administered rituximab and traditional Chinese medicine, respectively. Additionally, 63.4 and 43.1% of patients reported participating in few or no social activities and being out of work because of the disease. To be noted, 76.4% of patients reported suffering from negative emotions, with the most frequent being worry (60.2%), with 20.3% of patients experiencing suicidal thoughts. The inability to work and participating in few or no social activities due to NMOSD were two determinants of experiencing negative emotions (*P*_work_ = 0.03, OR_work_ = 3.34; *P*_socialactivities_ = 0.02, OR_socialactivities_ = 3.19).

**Conclusion:** This study reported patient perspectives on NMOSD in North China, whereby demonstrating that the inability to work and participating in few or no social activities due to NMOSD rather than the physical impairment caused by the disease, was directly associated with patients experiencing negative emotions. This insight offers potential ways to manage patients' negative emotions by enhancing family and social support and facilitating active employment.

## Introduction

Neuromyelitis optica spectrum disorder (NMOSD) is an inflammatory demyelinating disease of the central nervous system (CNS), characterized by preferential lesions in the optic nerve, spinal cord, and area postrema (1, 2). The highest incidence of NMOSD has been reported in the Afro-Caribbean region (0.73/100,000 person years), and the lowest incidence has been reported in Australia and New Zealand (0.037/100,000 person years) (3). In China, epidemiological data suggest that the incidence of NMOSD per 100,000 person years is 0.278 (4). The differential incidence of NMOSD indicates that genetic and environmental factors play a key role in developing this disorder (5). Further, the presence of AQP4-IgG in patients' serum and cerebrospinal fluid (CSF) confirms that NMOSD is an autoimmune disease mediated mainly by humoral immunity (6, 7). Although AQP4-IgG plays a pathogenic role in NMOSD, AQP4-IgG titers seem to fail to predict the disease course (8, 9). Moreover, the unpredictive recurrence of the disease not only results in cumulative neurological deficits involving paresthesia and visual and walking impairment (10, 11), but also leads patients to suffer from emotional or psychological disorders, which together significantly influence the quality of life for them and their families (12, 13). A study of 210 primarily AQP4-IgG-positive NMOSD patients from 25 provinces across China reported that NMOSD worsened both the physical and emotional health of patients and imposed a great negative impact on their quality of life (14). However, a study involving 193 patients with NMOSD from North America saw two-thirds of patients reporting that the disease had a strong negative impact on their physical health, whereas their emotional well-being was relatively unimpaired, on average (15). These differences in findings indicate that although NMOSD causes severe neurological dysfunctions, the psychological resilience of patients may be differentiated between ethnicities with different genetic and environmental backgrounds. Notably, the physical and emotional disorders of patients with NMOSD often lead to certain high-risk behaviors, such as self-harm and suicide (16). A more recent study exploring mortality and causes of death among 569 Chinese patients with NMOSD indicated that 8.3% of patients died due to suicide during a 10-year follow-up (17). Another study that evaluated the psychopathological profile and suicidality in an Argentinean NMOSD cohort illustrated that the prevalence of psychiatric disease was 45%, with 30% of patients attempting suicide at least once (16). Therefore, a comprehensive assessment of patients' physical and emotional conditions in certain regions or ethnicities is necessary to propose region-specific management to improve the quality of life for patients and reduce the incidence of high-risk behaviors.

Although the demographic and clinical features of patients with NMOSD had been reported in several regions and ethnicities worldwide (18–21), to our knowledge, there had been no study using patient-reported data to conduct a synthesized evaluation of the physical, emotional, medical, and socioeconomic status of NMOSD patients in North China. Therefore, in our study, we performed a cross-sectional survey in a single center of North China, to help shed light on modifying the personal and clinical care for patients with NMOSD, which in turn will help improve their physical and emotional health and quality of life.

## Methods

### Study Design

This study used a cross-sectional survey design based on an established questionnaire from the Multiple Sclerosis Patient Survival Report 2018, which was the first Chinese nationwide survey to reveal the survival status of patients with multiple sclerosis (MS) in China including five aspects: basic characteristics of patients, disease characteristics, diagnosis and treatment, psychological burden, and economic burden (22) (Supplementary Material). The implementation of the present survey was approved by the Research Ethics Committee of the Second Hospital of Hebei Medical University (Ref. no. 2018-P055).

### Participants

A total of 123 patients with NMOSD who were treated in the Department of Neurology at the Second Hospital of Hebei Medical University were enrolled in this survey, with informed consent obtained from each participant or his/her legal guardian. The implementation period of this survey was from August 2018 to 2019. All patients fulfilled the 2015 International Consensus Diagnostic Criteria for Neuromyelitis Optica Spectrum Disorders. Patients were given the option of completing the survey with the assistance of a relative, friend, or caregiver if their education status or physical impairments precluded independent participation. Patients with severe dementia, developmental delay, mental abnormalities, chronic kidney failure, tumors, liver cirrhosis, and any diseases or conditions that the investigator considered inappropriate for participation in this survey were excluded. Aside from these factors, there were no sex, age, or other additional restrictions when patients were enrolled.

### Survey Items

This survey was based on an established questionnaire from the Multiple Sclerosis Patient Survival Report 2018. The survey instrument was implemented either by telephone or *via* face-to-face communication to record the following items: basic patient characteristics (sex, current age, marital status, fertility status, and education status); disease characteristics (date of disease onset, symptoms at disease onset, attack history, symptoms during the course of the disease, medical consultation, and diagnostic history); treatment information (medications during the relapse and remission phase, healthcare expenditure, concerns regarding the current treatment); and emotional and socioeconomic conditions (emotional status, employment status, participation in social activities, concerns regarding the disease). The category of social activities included religious meetings; social clubs or leisure, cultural, or sports groups; alumni societies, societies for people from the same hometown, or family councils; and volunteer groups, political parties, non-government organizations, or interest groups.

Each patient data set was assessed for quality, internal consistency, and completeness with access limited to qualified study personnel to ensure security.

### Statistical Analyses

The Shapiro–Wilk Normality Test was performed for the Normal Distribution Test. If the variables were in accordance with normal distribution, they were presented as mean ± standard deviations (SD), and an independent sample *t*-test was used for comparison in two groups. If not in accordance with normal distribution, the variables were described as median (interquartile range), and the Mann-Whitney *U*-Test was used for comparison between the two groups. To be noted, the annual EDSS increase calculated as EDSS at last follow / disease duration (years) was used to assess the annualized disease exacerbation in this study. The binary logistic regression analysis was conducted to define the significant determinants of certain physical or emotional characteristics of patients, setting the “disease course (monophasic course vs. recurrent course),” “annual EDSS increase (annual EDSS increase < 1 vs. annual EDSS increase≥1,” “attack-prevent treatment (no vs. yes),” or “negative emotions (absent vs. present)” as the dependent variable and some demographic, clinical and socioeconomic data as independent variables (**Tables 2–5**). All the categorical data were analyzed by the Chi-square Test. Due to the exploratory nature of the study, no a priori sample size calculation was made without adjustment for multiple testing. A threshold of *P* < 0.05 was considered to indicate statistical significance. All datasets were analyzed using the SPSS 23.0 software.

## Results

### Demographics and Clinical Characteristics

**Figure 1:**
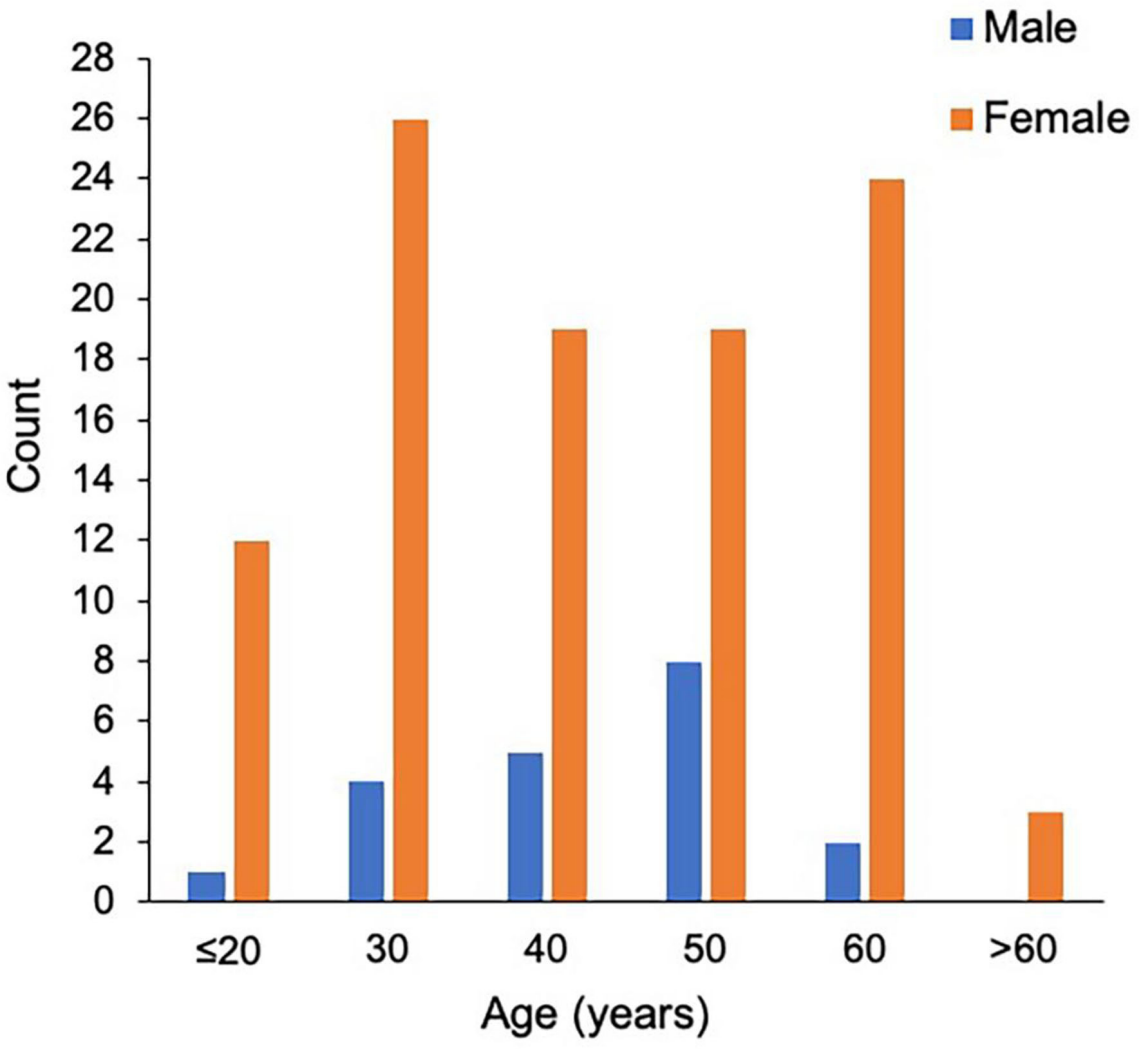
The distribution of age at onset in male and female patients with NMOSD.

The study population consisted of 123 patients with NMOSD, with the median age at disease onset being 38 years old and the female to male ratio being 5.2:1. Although the median age at onset was not significantly different, the male patients appeared to have a peak onset age of 40–50 years old, and in female cohorts there were two peaks at 20–30 and 50–60 years old (Figure 1). There were 97 patients (78.9%) who were seropositive for AQP4-IgG, and the proportion of female patients among seropositive patients was significantly higher than that among the seronegative cohort (88.7 vs. 63.6%, *P* < 0.05). The median disease duration was 3 years and the median Expanded Disability Status Scale (EDSS) score was 2.0. Among patients with disease duration ≥1 year, 72.2% were with relapse disease course, corresponding to an annual relapse rate (ARR) of 0.8. The median time between onset and first recurrence was 11 months, with 49.0% of patients experiencing a second episode within 1 year following disease onset (Table 1). Additionally, the results of logistic regression analysis demonstrated the following: there is no evident association between the factors of sex, age at onset, serostatus of AQP4-IgG, or symptoms at onset and the disease course (Table 2); disease onset in elderly (>50 years) patients is a risk factor of annual EDSS increase ≥1 as compared with disease onset in younger (< 30 years) patients [*P* = 0.001, OR = 7.83, 95%CI (2.24–27.40)] (Table 3).

**Table 1:** Summary of the demographic and clinical data of patients with NMOSD.

**Sex**	
Male (%)	20.0 (16.3)
Female (%)	103.0 (83.7)
Current age (years)^††^	43.0 (32.0–54.0)
Married (%)	111.0 (90.2)
Procreated (%)	100.0 (81.3)
**Education status**	
Elementary school (%)	24.0 (19.5)
Middle school (%)	45.0 (36.6)
High school (%)	15.0 (12.2)
University or above (%)	39.0 (31.7)
Age at onset (years)^††^	38.0 (27.0–50.0)
Disease duration (year)^††^	3.0 (1.0–5.0)
**AQP4-IgG**	
Positive (%)	97.0 (78.9)
Negative (%)	11.0 (8.9)
Unknown result (%)	9.0 (7.3)
Not checked (%)	6.0 (4.9)
EDSS^††^	2.0 (1.0–3.5)
Disease course^*^	
Monophasic course (%)	30.0 (27.8)
Disease duration (year)^††^	1.0 (1.0–2.3)
Relapsing-remitting course (%)	78.0(72.2)
Disease duration (year)^††^	5.0 (2.4–7.0)
Annual relapse rate (ARR)^††^^*^	0.8 (0.5–1.0)
Annual EDSS increase^††^^*^	0.7 (0.2–1.5)
Time between onset and first recurrence (month)^††^	11.0 (5.0–24.0)
**Medical consultation after the first symptom**	
Immediately (<72 h, %)	81.0 (65.9)
Not immediately (≥72 h, %)	42.0 (34.1)
Time of medical consultation delay (day)^††^	30.0 (15.0–60.0)
Diagnostic delay (month)^††^	6.0 (2.0–24.0)

*NMOSD, neuromyelitis optica spectrum disorders; EDSS, Expanded Disability Status Scale; AQP4, aquaporin-4; IQR, interquartile range.*

†† *Indicating median (IQR)*;

* *Patients with disease duration ≥ 1 year were assessed these indexes*.

**Table 2:** Binary logistic regression analysis of factors associated with disease course (Monophasic course vs. Recurrent course) in NMOSD.

**Variables**	**Odds ratio**	**95% CI**		***P*** **-value**
		**Lower**	**Upper**	
Sex (Male/Female)	0.64	0.16	2.58	0.53
**Age at onset**				
Age at onset (<30 y)				
Age at onset (30–50 y)	0.97	0.31	3.03	0.96
Age at onset (>50 y)	0.87	0.24	3.23	0.84
AQP4-IgG status (Neg/Pos)	0.73	0.14	3.71	0.70
**Symptoms at onset (Absent/Present)**				
Visual impairment	1.52	0.40	5.78	0.54
Limb weakness	0.38	0.09	1.65	0.20
Paresthesia	0.43	0.09	2.04	0.29
Bladder or bowel dysfunction	2.55	0.70	9.27	0.16
Balance disturbance	0.45	0.11	1.76	0.25
Muscle stiffness and spasm	1.67	0.33	8.40	0.54
Dysphasia	1.48	0.08	26.26	0.79
Nausea, vomiting, hiccups	3.97	0.26	61.80	0.33
Fatigue	0.65	0.16	2.72	0.56
Pain	1.54	0.40	5.88	0.53
Cognition impairment	0.30	0.03	3.18	0.32

*NMOSD, neuromyelitis optica spectrum disorders; y, years; AQP4, aquaporin-4; Neg, negative; Pos, positive*.

**Table 3:** Binary logistic regression analysis of factors associated with annual EDSS increase (annual EDSS increase < 1 vs. annual EDSS increase ≥1) in NMOSD.

**Variables**	**Odds ratio**	**95% CI**		***P*** **-value**
		**Lower**	**Upper**	
Sex (Male/Female)	1.92	0.74	5.62	0.18
Age at onset				
Age at onset (<30 y)				
Age at onset (30–50 y)	1.67	0.57	4.94	0.35
Age at onset (>50 y)	7.83	2.24	27.40	**0.001**
AQP4-IgG status (Neg/Pos)	3.33	0.67	16.64	0.14
Preventative treatment (Y/N)	0.73	0.26	2.07	0.56

*NMOSD, neuromyelitis optica spectrum disorders; EDSS, Expanded Disability Status Scale; y, years; AQP4, aquaporin-4; Neg, negative; Pos, positive; Y, yes; N, no. Bold value indicated significant difference*.

**Figure 2:**
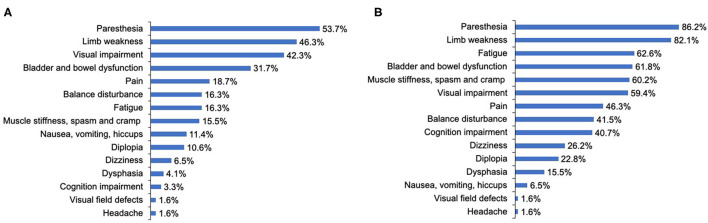
The clinical symptoms at disease onset and throughout the disease course of patients with NMOSD. **(A)** The clinical symptoms at disease onset. **(B)** The clinical symptoms throughout the disease course. Cognition indicated attention, concentration, recognition, judgment, and memory.

The most common initially presenting symptoms in our study were paresthesia (53.7%), limb weakness (46.3%), and visual impairment (42.3%) (Figure 2A). Among 123 patients, 65.9% immediately (< 72 h) sought medical consultation following the appearance of initial symptoms. The delay among patients not seeking immediate medical consultation was 30 (15–60) days. Moreover, the department where patients most frequently visited initially was Neurology (39.8%), Ophthalmology (33.3%), General Internal Medicine (24.4%), and Orthopedics (2.4%). A total of 63.4% of participants were initially diagnosed with a condition other than NMOSD, and of these patients, 43.6% did not receive a definitive diagnosis and 56.4% were diagnosed with other diseases. The median delay between initial symptom episode to receiving an accurate diagnosis of NMOSD was 6 months.

During the course of the disease, over 80% of patients reported that they had experienced symptoms of paresthesia (86.2%) and limb weakness (82.1%). The proportion of patients reporting visual impairment throughout the course of the disease increased slightly compared with that at disease onset, from 42.3 to 59.4%. As for other neurological dysfunction symptoms, only 16.3, 15.5, 31.7% of patients reported balance disturbance; muscle stiffness, spasm, and cramp; and bladder and bowel dysfunction at onset, respectively, but these proportions increased dramatically to 41.5, 60.2, and 61.8%, respectively, throughout the course of the disease. Further, cognition impairment (attention, concentration, recognition, judgment, and memory), fatigue, and pain were reported by merely 3.3, 16.3, and 18.7% of patients, respectively, at disease onset, whereas during the course of the disease, these proportions increased significantly to 40.7, 62.6, and 46.3%, respectively (Figure 2B).

### Treatment

**Figure 3:**
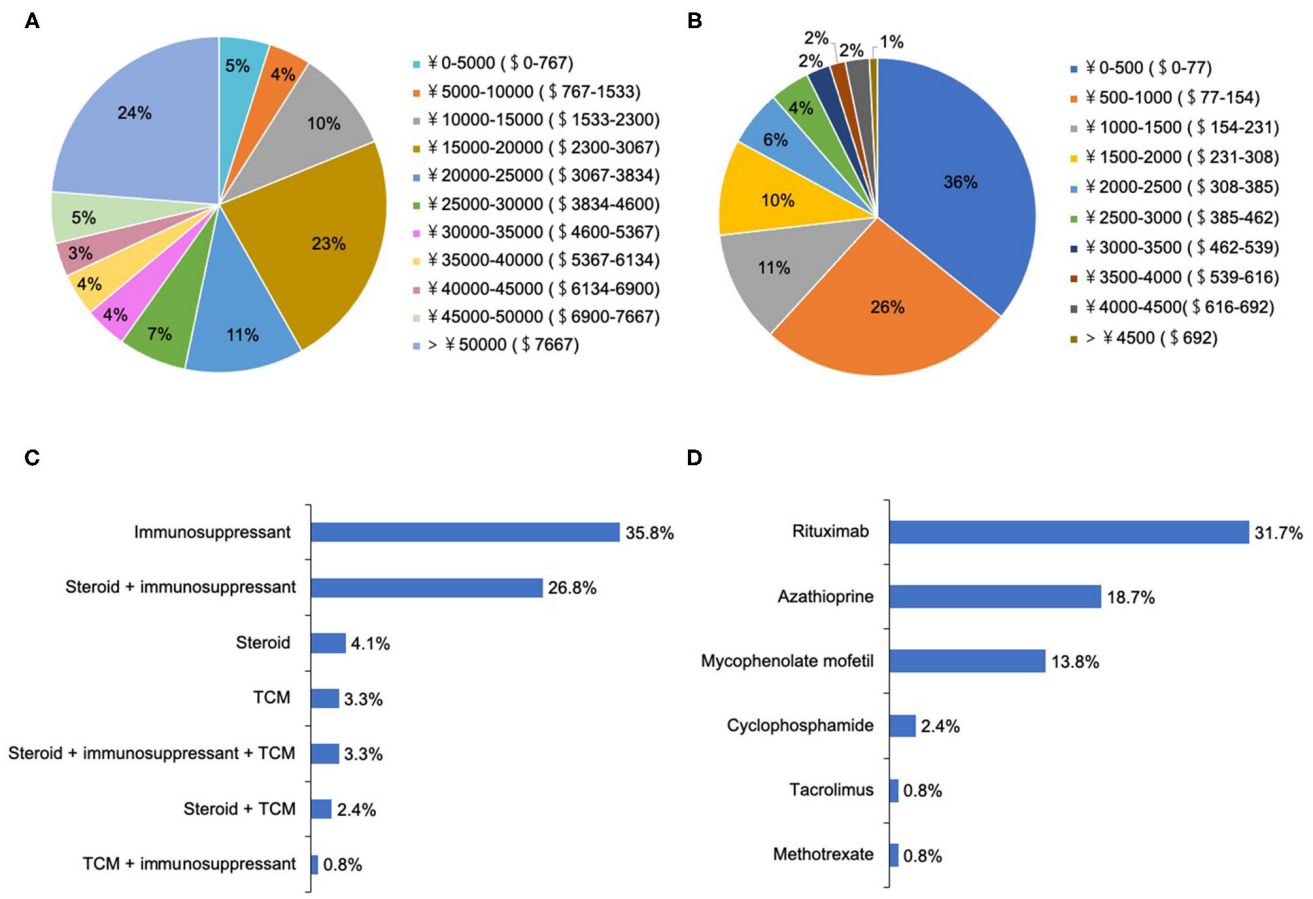
The treatment selections and expenditure for patients with NMOSD. **(A)** The hospitalization expenditure per recurrence (ɤ, CNY; $, USD). **(B)** The medical expenditure in the remission phase per month (ɤ, CNY; $, USD). **(C)** The medical selections in the remission phase. **(D)** The choices of immunosuppressants in the remission phase.

A total of 91.9% of patients chose to be hospitalized for each recurrence, where they received the following treatments: intravenous methylprednisolone (IVMP) (98.4%), IVMP and gamma-globulin (35.8%), and plasma exchange (PLEX, 6.5%). There were two peaks for hospitalization expenditure per recurrence: 15,000–20,000 CNY ($2,300– $3,067 USD) and >50,000 CNY (>$7,667 USD) (Figure 3A).

During remission, 76.4% of patients underwent attack-prevention therapies including immunosuppressants (IS), low-dose steroids, and Traditional Chinese Medicine (TCM) therapy. The results of logistic regression analysis demonstrate that patients with fatigue were 2.78 times more likely to accept these preventive treatments than those without (*P* = 0.03), whereas a patient's sex, age at onset, education level, employment status, disease course, EDSS, and other clinical symptoms were not clear factors in whether they chose to receive preventive treatment (Table 4). Time from NMOSD diagnosis to initiation of preventive therapy was ≤ 1 month (57.5%), 1–3 months (8.5%), 3–6 months (8.5%), 6–2 months (7.5%), 12–24 months (2.1%), and ≥24 months (16.0%). The medication selected by patients during remission included IS alone (35.8%), low-dose steroid and IS (26.8%), and steroid alone (4.1%) (Figure 3C). Patients treated with IS were administered rituximab (RTX, 31.7%), azathioprine (AZA, 18.7%), mycophenolate mofetil (MMF, 13.8%), cyclophosphamide (CYC, 2.4%), tacrolimus (TAC, 0.8%), and methotrexate (MET, 0.8%) (Figure 3D).

**Table 4:** Binary logistic regression analysis of factors associated with receiving attack-prevent treatment (No vs. Yes) in NMOSD.

**Variables**	**Odds ratio**	**95% CI**		***P*** **-value**
		**Lower**	**Upper**	
Sex (Male/Female)	0.15	0.03	0.90	0.06
**Age at onset**				
Age at onset (<30 y)				
Age at onset (30–50 y)	0.49	0.13	1.85	0.29
Age at onset (>50 y)	0.71	0.14	3.73	0.69
**Education status**				
Elementary school				
Middle school	1.43	0.35	5.86	0.62
High school	1.34	0.16	11.23	0.79
University or above	0.81	0.15	4.44	0.81
Disease course (Monophasic/Recurrent)	2.41	0.75	7.75	0.14
EDSS	0.97	0.72	1.31	0.85
**Symptoms in the entire disease course (Absent/Present)**				
Visual impairment	1.76	0.56	5.49	0.33
Limb weakness	2.60	0.54	12.48	0.23
Paresthesia	1.48	0.34	6.45	0.60
Bladder or bowel dysfunction	0.75	0.17	3.25	0.70
Balance disturbance	1.79	0.55	5.82	0.34
Muscle stiffness and spasm	0.43	0.11	1.61	0.21
Dysphasia	8.80	0.85	91.61	0.07
Nausea, vomiting, hiccups	0.49	0.06	4.19	0.51
Fatigue	2.78	1.12	6.93	**0.03**
Pain	1.23	0.40	3.85	0.72
Cognition impairment	0.49	0.16	1.52	0.22
Employment status	1.35	0.37	4.97	0.65

*NMOSD, neuromyelitis optica spectrum disorders; y, years; EDSS, Expanded Disability Status Scale. Bold value indicated significant difference*.

**Figure 4:**
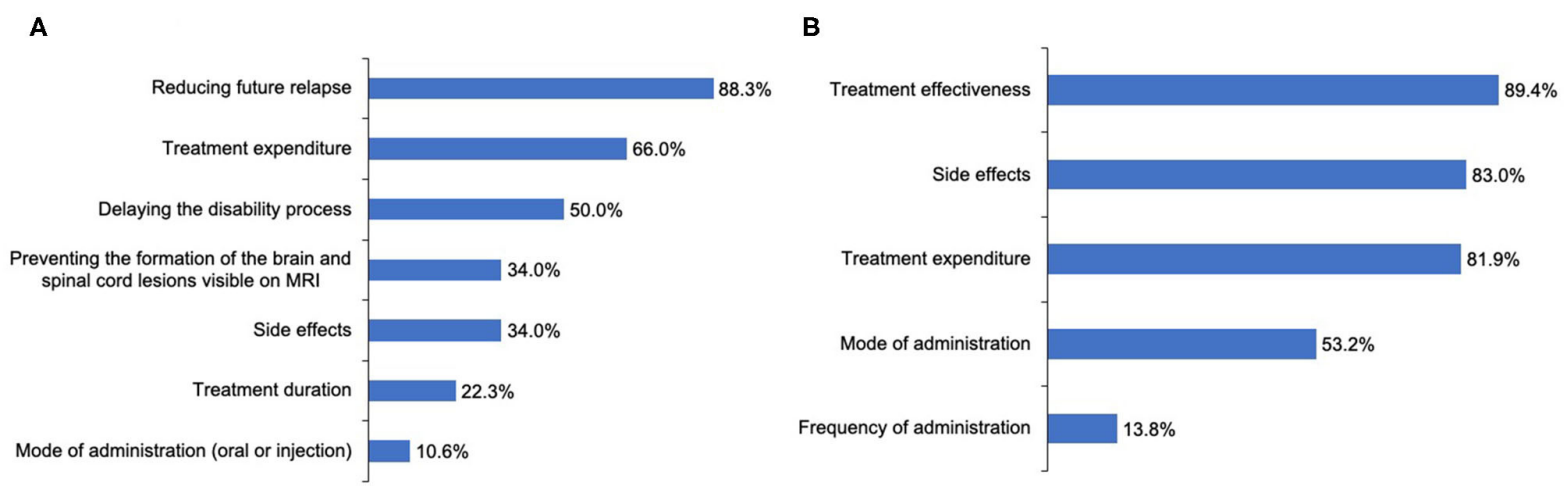
Concerns and aspects for improvement regarding the current preventive treatment for patients with NMOSD. **(A)** Patients' concerns about the current preventive treatment. **(B)** Aspects for improvement in the current preventive treatment reported by the patients.

Additionally, 10.6% of patients had ever accepted TCM treatment, and 3.3% were treated with TCM alone. Regarding the medical expenditure in the remission phase, more than half (62%) of patients reported their expenditure per month was < 1,000 CNY ($154 USD) (Figure 3B). In the 6 months preceding our survey, only 44.7% of patients underwent routine outpatient health follow-ups. The main concerns surrounding NMOSD treatment in remission among patients was reducing future relapse (83.3%), treatment expenditure (66.0%), and delaying the disability process (50%) (Figure 4A). Furthermore, when it came to present attack-prevention treatment, patients stated that improvements needed to be made with regard to treatment effectiveness (89.4%), side effects (83.0%), and treatment expenditure (81.9%) (Figure 4B).

### Socioeconomic and Emotional Conditions

Patients reported completing elementary school (19.5%), middle school (36.6%), high school (12.2%), and university or above (31.7%). Although nearly one-third of patients had completed university education or above, 89 patients (72.4%) were unemployed, 53 (59.6%) of whom reported incapacity to work due to the disease. Alternatively, 15 (12.2%) and 23 (18.7%) patients reported demanding unilateral or bilateral walking aids and family health nursing. Seventy-eight patients (63.4%) reported having few or no social activities after being diagnosed with NMOSD.

**Figure 5:**
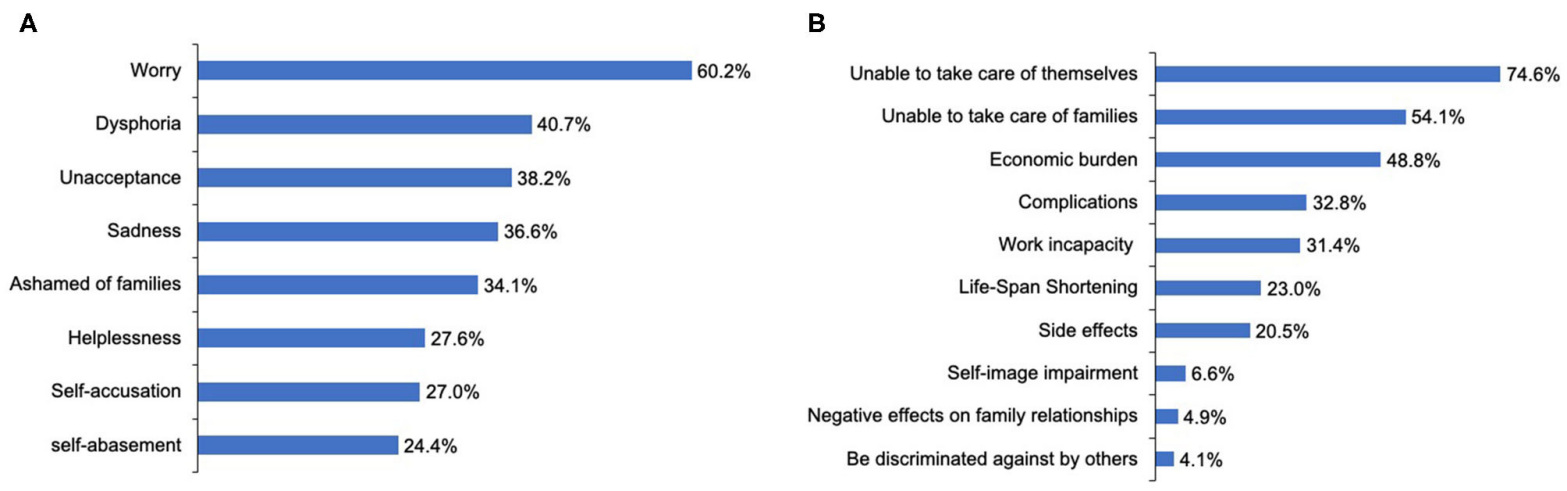
Emotional status of patients with NMOSD and their concerns regarding this disorder. **(A)** The negative emotions experiencing by the patients with NMOSD. **(B)** Patients' concerns about NMOSD.

During the course of the disease, 94 patients (76.4%) reported suffering from negative emotions, and 25 patients (20.3%) had suicidal thoughts, four of whom had ever committed suicide. The most frequent negative emotion reported by patients was worry (60.2%) (Figure 5A). Logistic regression analysis suggested that the inability to work and participation in few or no social activities due to NMOSD are two determinants of patients experiencing negative emotions [*P*_work_ = 0.03, OR_work_ = 3.34, 95% CI (1.09–10.19); *P*_socialactivities_ = 0.02, ORs_ocialactivities_ = 3.19, 95% CI (1.18–8.61)] (Table 5). With regard to the disease, patients reported their concerns primarily in aspects of being unable to take care of themselves (74.6%) or family members (54.1%), and disease-specific economic burdens (48.8%) (Figure 5B).

**Table 5:** Binary logistic regression analysis of factors associated with negative emotions (Absent vs. Present).

**Variables**	**Odds Ratio**	**95%CI**		***P*** **-value**
		**Lower**	**Upper**	
Sex (Male/Female)	3.60	0.64	20.20	0.15
**Age at onset**				
Age at onset (<30y)				
Age at onset (30–50y)	1.22	0.31	4.89	0.78
Age at onset (>50y)	0.51	0.08	3.17	0.47
**Education status**				
Elementary school				
Middle school	0.26	0.05	1.49	0.13
High school	0.20	0.03	1.59	0.13
University or above	1.11	0.13	9.33	0.92
Disease duration	1.02	0.85	1.23	0.83
Disease course (Monophasic/Recurrent)	1.96	0.47	8.16	0.36
EDSS	1.44	0.80	2.57	0.22
**Symptoms in the entire disease course (Absent/Present)**				
Visual impairment	1.10	0.30	4.05	0.88
Limb weakness	0.41	0.07	2.36	0.32
Paresthesia	1.99	0.36	11.06	0.43
Bladder or bowel dysfunction	0.91	0.18	4.74	0.91
Balance disturbance	0.86	0.22	3.34	0.83
Muscle stiffness and spasm	1.37	0.33	5.68	0.66
Dysphasia	2.30	0.34	15.47	0.39
Nausea, vomiting, hiccups				1.00
Fatigue	1.15	0.30	4.46	0.84
Pain	1.33	0.39	4.60	0.65
Cognition impairment	2.40	0.68	8.42	0.17
Social activities	3.19	1.18	8.61	**0.02**
Disease-related employment status	3.34	1.09	10.19	**0.03**

*NMOSD, neuromyelitis optica spectrum disorders; y, years; EDSS, Expanded Disability Status Scale.*

*Bold value indicated significant difference*.

## Discussion

The demographic characteristics and clinical profiles of the patients in this study were similar to those in other ethnicities reported by previous publications: NMOSD is more prevalent in females (20, 23–26), with AQP4-IgG detected in over half of NMOSD populations (20, 23, 25, 27). NMOSD patients often suffer from frequent relapse and severe neurological disabilities, with paresthesia, limb weakness, and visual impairment being the most common initial symptoms (15, 20, 23, 27, 28). In this study, disease onset in elderly patients was associated with a higher annual EDSS increase compared with disease onset in younger patients, which is similar to the findings of several other studies and may be due to the following aspects (24, 29–31). (1) In some previous reports, disease onset in elderly patients was often characterized by severe attacks such as longitudinally extensive transverse myelitis, which resulted in disability within a shorter time, thereby carrying a worse outcome (29). (2) During aging, there is a substantial decline in the ability to resist abnormal immune responses and inflammatory responses, as well as a corresponding decline in the generation of robust protective responses (32). (3) Disease onset in elderly patients often exhibits a less positive response to immunosuppressive therapy, and patients experience more side effects with more frequent and severe comorbidities (29, 30). The disease duration of patients with the relapsing–remitting course group was significantly longer than those with the monophasic course. This could be attributed to several reasons: (1) Patients with the monophasic disease had a relatively short follow-up period and the relapse may have occurred over time in some individuals. (2) Patients with the monophasic disease were diagnosed recently and may have received preventive therapy earlier due to current physicians' understanding of the importance of early preventive intervention for NMOSD. Alternatively, our study revealed that there is no significant association between the factors of sex, age at disease onset, serostatus of AQP4-IgG, or symptoms at onset and the disease course, which is consistent with the findings of a previous study (33). The diagnostic delay observed in our study was 6.0 (2.0 – 24.0) months, which seems to be shorter than that reported by another study (3.3 ± 6.3 years) (15), perhaps due to the rapid improvement of specimen transportation and standard antibody detection networks in North China during recent years.

In our study, 98.4% of patients were treated with IVMP and only 6.5% were treated with PLEX during hospitalization. The proportion of patients receiving PLEX was significantly lower than that in the USA (18.2%) and Latin America (62.1%) (15, 27). Furthermore, acute treatment with PLEX appears to be particularly helpful in NMOSD (34). Hence, the low application rate of PLEX therapy for patients in this study should arouse the attention of clinicians, and attach sufficient significance to the application of PLEX therapy in the future treatment of patients. Additionally, 76.4% of patients had received the attack-prevention treatment. In another study analyzing factors affecting the acceptance of immunotherapy for patients with MS in China, education status, patient understanding of the disease, and clinical symptoms all had a much higher probability of appearing in the treated group than in the untreated group (22). However, in our study, only patients with symptoms of fatigue were more likely to accept preventive treatments than those without, which may be partly due to the limited number of patients enrolled in our survey.

In terms of the medications applied in remission, 31.7% of patients selected RTX, which is a similar proportion to that in Korea (41%) but significantly lower than that in Germany (52.6%), Latin America (86.3%), and the USA (60.6%) (15, 27, 35, 36), perhaps partly owing to the high price of RTX. In our study, 10.6% of patients with NMOSD had ever received the TCM treatment, with the effectiveness of TCM in NMOSD treatment still needing to be confirmed *via* a series of large-scale studies in the future. Regarding the medical expenditure in the remission phase, 62% of patients reported that their expenditure per month was < 1,000 CNY ($154 USD), which was significantly lower than that in the USA ($479 USD) (15). This may be because these patients did not receive routine outpatient health follow-ups and usually selected relatively inexpensive steroid or non-specific immunosuppressants for their remission treatment.

In this study, cognition impairment, fatigue, and pain were reported by 40.7, 62.6, and 46.3%, respectively, throughout the course of the disease. Similar to these findings, previous studies reported that more than half of patients with NMOSD suffered from the above symptoms in different regions among different ethnicities (12, 16, 37, 38). To be noted, 76.4% of patients reported suffering from negative emotions in our current study. This is consistent with the findings of another study on NMOSD patients from 25 provinces of China, which revealed that NMOSD worsened both the physical and emotional health of patients but is inconsistent with the findings of a study of North American NMOSD patients, which reported NMOSD as having a strong negative impact on patients' physical health but not on patients' emotional well-being (14, 15). These findings indicate that different regions and corresponding social cultures may result in a different ability to cope with the pressure and problems brought about by the physical disabilities caused by NMOSD. Traditional Chinese culture advocates introversion and reserved expression of health-related concerns, which leads a certain number of Chinese people to be unaccustomed to talk about the distress caused by disease, thus these patients tend to gain less outside support (39).

Moreover, in our study, 63.4 and 43.1% of patients reported participating in few or no social activities and being out of work because of the disease. These results were similar with the findings of other studies involving patients from China, the Middle East, and North America, suggesting that patients with NMOSD across different ethnicities and regions are often faced with unemployment and reduced participation in social activities (14, 15, 40). Further, the inability to work and participation in few or no social activities, rather than physical impairment or discomfort symptoms (i.e., visual impairment, walking difficulty, pain, and fatigue) was directly associated with having negative emotions. These findings are somewhat different from previous reports. In those studies, the inability to work and the financial burdens, as well as physical impairments (i.e., pain, fatigue, and sexual function impairment) had negatively impacted the emotional health of patients (12). The finding in our study could be attributed to several reasons: (1) The results of this study were obtained from patient-reported data rather than through a systematic scale for symptoms and emotional health evaluation applied by others; (2) Geographical specificity: The economy of Hebei Province in China is underdeveloped, with the capita disposable income per person in 2018 being 23,446 CNY ($3,620 USD) (41). In our study, economic factors were also one of the main concerns of patients in this region with regard to both treatment and disease. Therefore, the reduced income caused by unemployment and the overall economic burden were more likely to cause patients to have negative emotions. Moreover, people in North China are more evidently affected by traditional Chinese culture. Many patients are unwilling to talk publicly about the discomfort and negative emotions caused by the disease. The above consideration affects patients' social activities and significantly reduces the social support they may have received. Therefore, this finding demonstrates that enhancing socioeconomic support networks and facilitating active employment is an effective strategy for coping with health-related challenges and negative emotions for patients with NMOSD in North China.

This study still has several limitations: First, due to the rarity of NMOSD, the number of patients enrolled in the present survey was limited. Second, most of the data were reported by the patients or their families, and no systematic scale for fatigue, pain, emotional health, and quality of life assessment was applied, which may lead to certain recall bias and impede the objective quantification of results. With that said, this patient-reported information enabled our study to directly reflect patient perspectives on NMOSD. The conclusions of our study should be considered with caution, and further studies with larger accounts of patients, employing systematic scales for fatigue, pain, emotional health, and quality of life assessments are needed to confirm the findings of our study.

## Conclusions

Our study reported patient perspectives on NMOSD in North China, and demonstrated that the inability to work as well as participating in few or no social activities is directly associated with patients experiencing negative emotions. This finding provides novel insight into potentially modifiable aspects for helping patients to deal with their negative emotions, including enhancing family and social support and facilitating active employment.

## Data Availability Statement

The raw data supporting the conclusions of this article will be made available by the authors, without undue reservation.

## Ethics Statement

The studies involving human participants were reviewed and approved by Research Ethics committee of the Second Hospital of Hebei Medical University. The patients/participants provided their written informed consent to participate in this study.

## Author Contributions

ZJ contributed with conception, design, acquisition, analysis and interpretation of data, statistical analysis, drafting, and revision of the manuscript. XD and SS contributed with analysis and interpretation of data and revision of the manuscript. RG and LZ contributed with revision of the manuscript. JL and BL contributed with study supervisor. All authors contributed to the article and approved the submitted version.

## Funding

This study was supported by the 2020 Hebei Medical Science Research Project Plan of China (Grant No. 20200040) and the National Natural Science Foundation of China (Grant No. 81873759).

## Conflict of Interest

The authors declare that the research was conducted in the absence of any commercial or financial relationships that could be construed as a potential conflict of interest.

## Publisher's Note

All claims expressed in this article are solely those of the authors and do not necessarily represent those of their affiliated organizations, or those of the publisher, the editors and the reviewers. Any product that may be evaluated in this article, or claim that may be made by its manufacturer, is not guaranteed or endorsed by the publisher.
