# Supplementary material for: Physical, Emotional, Medical, and Socioeconomic Status of Patients With NMOSD: A Cross-Sectional Survey of 123 Cases From a Single Center in North China
Source: Front Neurol. 2021 Sep 8;12:737564. doi: 10.3389/fneur.2021.737564 (PMC8455822; doi:10.3389/fneur.2021.737564)
Supplement: Supplementary file 1 [file Table_1.DOCX]

**Survey Questionnaire**

1. Patient ID

2. Patient name

3. Sex

○ *Male*

○ *Female*

4. Birthdate

5. Telephone/ Cell phone Number

6. Email address

7. Marital status

○ *Unmarried*

○ *Married*

8. Fertility status

○*Unprocreated*

○*Procreated*

9. Education status

○*Elementary school*

○*Middle school*

○*High school*

○*University or above*

10. Age at disease onset

11. Fertility status at disease onset

○*Unprocreated*

○*Procreated*

12. Date of disease onset or initial presenting symptoms

13. Date of initial medical consultation

14. Date of diagnosis with NMO/NMOSD

15. Date of the latest attack preceding this survey

16. Time between onset and first recurrence () months

17. Attack number within the latest 1 year preceding this survey

18. Attack number within the latest 2 years preceding this survey

19. Total attack number throughout the disease course

20. Have you heard of “NMOSD” before being diagnosed?

○*Yes*

○*No*

21. Have you immediately (<72h) sought the medical consultation after the appearance of the first symptoms?

○*Yes*

○*No*

22. You initially sought the medical consultation at the Department of

○*Neurology*

○*Ophthalmology*

○*General Internal Medicine*

○*Orthopedics*

○*Others (Please clarify)*

23. Were you diagnosed with NMOSD at the initial medical

consultation

○*Yes*

○*No*

24. If you were not diagnosed with NMOSD at the initial medical consultation, what was the diagnosis?

○*Undiagnosed with any definitive disease*

○*Diagnosed with another disease*

25. Time between initial presenting symptoms and diagnosed with NMOSD () months.

26. The initial presenting symptoms were (Multiple choices):

○*Paresthesia (*i.e., *numbness, tingling, or burning)*

○*Limb weakness or walking difficulty*

○*Visual impairment*

○*Bladder or bowel incontinence*

○*Pain*

○*Balance disturbance or unsteady gait*

○*Fatigue*

○*Muscle stiffness, spasm, and cramp*

○*Nausea, vomiting, hiccups*

○*Diplopia*

○*Dizziness*

○*Dysphasia*

○*Cognition (attention, concentration, recognition, judgment, and memory) impairment*

○*Headache*

27. The symptoms had ever presented throughout the disease course (Multiple choices):

○*Paresthesia (i.e., numbness, tingling, or burning)*

○*Limb weakness or walking difficulty*

○*Visual impairment*

○*Bladder or bowel incontinence*

○*Pain*

○*Balance disturbance or unsteady gait*

○*Fatigue*

○*Muscle stiffness, spasm, and cramp*

○*Nausea, vomiting, hiccups*

○*Diplopia*

○*Dizziness*

○*Dysphasia*

○*Cognition (attention, concentration, recognition, judgment, and memory) impairment*

○*Headache*

28. Do you need unilateral or bilateral walking aids at present (i.e., canes, crutches, walkers, and rollators)?

○*Yes*

○*No*

29. Do you need family health nursing at present?

○*Yes*

○*No*

30. Do you chose hospitalization for each recurrence?

○*Yes*

○*No*

31. The treatment or drug you had ever receive in the attack phase was/were

○*Intravenous methylprednisolone*

○*Plasma exchange*

○*γ-globulin*

○*Others (Please clarify......)*

32. Hospitalization expenditure per recurrence（USD:CYN = 1:6.52）

○*0-5000**CNY (＄0-767 USD)*

○*5000-10000 CNY (＄767-1533* *USD)*

○*10000-15000 CNY (＄1533-2300* *USD)*

○*15000-20000 CNY (＄2300-3067* *USD)*

○*20000-25000 CNY (＄3067-3834* *USD)*

○*25000-30000 CNY (＄3834-4600* *USD)*

○*30000-35000 CNY (＄4600-5367 USD)*

○*35000-40000 CNY (＄5367-6134 USD)*

○*40000-45000 CNY (＄6134-6900 USD)*

○*45000-50000 CNY (＄6900-7667 USD)*

○*＞50000 CNY (＄＞7667 USD)*

33. Attack preventive treatment selection in the remission phase

*○ No treatment*

*○Low-dose steroid (LDS)*

*○Immunosuppressants (IS)*

*○ LDS plus IS*

*○Traditional Chinese Medicine (TCM)*

*○LDS plus TCM*

*○IS plus TCM*

○LDS plus IS plus TCM

34. Immunosuppressants selections (Multiple choices)

○*Rituximab*

○*Azathioprine*

○*Mycophenolate mofetil*

○*Cyclophosphamide*

○*Tacrolimus*

○*Methotrexate*

○*Others (Please clarify)*

35. Time from NMOSD diagnosis to initiation of preventive therapy

○*≤1 month*

○*1-3 month*

○*3-6 month*

○*6-12 month*

○*12-24 month*

○*≥24 month*

36. The medical expenditure in the remission phase including prescription medicine, travel to clinical care, medical supplies, and caregiver or service per month was : （USD:CYN = 1:6.52）

○*0-500 CNY (＄0-77* *USD)*

○*500-1000 CNY(＄77-154* *USD)*

*○1000-1500 CNY (＄154-231* *USD)*

○*1500-2000 CNY(＄231-308 USD)*

○*2000-2500 CNY(＄308-385* *USD)*

○*2500-3000 CNY(＄385-462* *USD)*

○*3000-3500 CNY (＄462-539 USD)*

○*3500-4000 CNY (＄539-616* *USD)*

○*4000-4500 CNY(＄616-692* *USD)*

○>*4500 CNY (＄>692* *USD)*

37. Did you go to the routine outpatient follow-up for the disease in the latest 6 months preceding this survey?

○*Yes*

○*No*

38. What are the three most important concerns when you consider

receiving attack preventive therapy for this disease?

○*Reducing future relapse*

○*Treatment expenditure*

○*Delaying the disability process*

○*Preventing the formation of the brain and spinal cord lesions visible on MRI*

○*Side effects*

○*Treatment duration*

○*Mode of administration (oral or injection)*

39. As for the attack preventive therapy, improvements needed to be made mainly in aspects of (Choose up to 3 items)

○*Treatment effectiveness*

○*Side effects*

○*Treatment expenditure*

○*Mode of administration*

○*Frequency of administration*

○*Others (Please clarify)*

40. Since suffering from NMOSD, have you experienced any of the following negative emotions continuously? (Multiple choice)

○*Worry*

○*Dysphoria*

○*Unacceptance*

○*Sadness*

○*Ashamed of families*

○*Helplessness*

○*Self-accusation*

○*Self-abasement*

○*Not experienced any negative emotion*

41. Since suffering from NMOSD, have you ever had suicidal thoughts?

○*Yes*

○*No*

42. Since suffering from NMOSD, have you ever committed suicide?

○*Yes*

○*No*

43. Did you inform your relatives or friends that you were diagnosed with NMOSD?

○*Yes*

○*No*

44. When you had some negative emotions, were you willing to share these with your friends or relatives?

○*Yes*

○*No*

45. Was your participation in social activities affected due to the disease?

○*Unaffected or almost unaffected*

○*Obviously affected / having little or no social activity*

46. Are you currently at work or employed?

○*Yes*

○*No*

47. If your answer to Q45 was “no”, were you out of work due to the disease?

○*Yes*

○*No*

48. Regarding the disease, which three issues were you most concerned about?

○*Unable to take care of themselves*

○*Unable to take care of families*

○*Economic burden*

○*Complications*

○*Work incapacity*

○*Life-Span Shortening*

○*Side effects*

○*Self-image impairment*

○*Negative effects on family relationships*

○*Be discriminated against by others*
